# Effects of prophylactic nalbuphine on emergence agitation and postoperative pain in pediatric patients undergoing ENT surgery with sevoflurane anesthesia

**DOI:** 10.3389/fped.2024.1353027

**Published:** 2024-09-17

**Authors:** Wendong Han, Jingjie Cai, Wangping Zhang, Rong Wei, Yan Jiang

**Affiliations:** ^1^ Department of Anesthesiology, Shanghai Children’s Hospital, School of Medicine, Shanghai Jiao Tong University, Shanghai, China; ^2^ Department of Anesthesiology, Women and Children’s Hospital of Jiaxing University, Jiaxing, China

**Keywords:** nalbuphine, agitation, sevoflurane, general anesthesia, children

## Abstract

**Background:**

Emergence agitation (EA) is a common complication in the pediatric population. This study aimed to investigate the effect of the prophylactic nalbuphine on EA in pediatric patients receiving sevoflurane anesthesia.

**Methods:**

The children undergoing ear, nose, and throat (ENT) surgery were administered 0.2 mg/kg nalbuphine (the nalbuphine group) or the same volume of normal saline (the control group) 5 min before the end of the surgery. The extubating time, time to eye-opening and duration of the post-anesthesia care unit (PACU) were recorded. Heart rate and blood pressure were monitored before and 5 min after nalbuphine administration. Pain was assessed using Face Legs Activity Cry and Consolability (FLACC) scales, and the drug-related postoperative complications (e.g., EA, delayed awakening, nausea and vomiting, and respiratory depression) were recorded.

**Results:**

One-hundred and thirty pediatric patients were randomly divided into nalbuphine and control groups (*n* = 65). The nalbuphine group showed a significantly lower incidence of EA than the control group (20% vs. 46.2%, *P *= 0.002). No significant differences between the two groups were observed in heart rate and blood pressure 5 min after nalbuphine administration (*P* > 0.05). No significant differences were observed between the two groups regarding extubating time, time to eye-opening, and duration of PACU. The FLACC scales demonstrated lower values in the nalbuphine group than in the control group during the initial 4 h after the surgery. However, the FLACC scales showed similar values between 5 and 12 h after the surgery.

**Conclusions:**

In summary, the results of this study demonstrated that prophylactic natbuphine could minimize the incidence of EA in pediatric patients following ENT surgery without increasing the extubating time and PACU duration.

**Clinical Trial Registration:**

http://www.chictr.org.cn, identifier [ChiCTR2300070046].

## 1 Introduction

Sevoflurane is an inhaled anesthetic with advantages such as the rapid onset of action, minimal respiratory irritation, and quick metabolism (1). It is commonly used in young children who are having anesthesia, but it is also known to produce emergence agitation (EA), which is a common side effect of general anesthesia (2, 3). The incidence of EA with sevoflurane has been reported to be 50%–80% (4, 5), and has considerably increased among pediatric patients undergoing ear, nose, and throat (ENT) surgery, especially in those receiving inhalation general anesthesia (6). EA can easily contribute to incision bleeding, venous catheter detachment, reflux aspiration, falling out of bed, and an increase in nursing difficulty (7). Therefore, it is imperative to implement appropriate precautions to avoid EA in children who have undergone this specific surgical procedure.

The pathogenesis of EA is not clearly understood, and it is closely associated with the use of inhalational anesthetics, operative type, age, pre-existing anxiety of children and postoperative pain (3). The treatment of EA is mediated through some drug interventions. Prior research has demonstrated the efficacy of propofol, benzodiazepine medications, *α*2 agonists, and opioid drugs in preventing pediatric EA. However, these medications are also linked to adverse side effects, including respiratory depression, delayed extubation, and longer stays in the post-anesthesia care unit (PACU) (6–8). Recent literature indicates that esketamine may decrease the incidence of EA in pediatric patients, but it is associated with nightmares (9).

Nalbuphine is a synthetic opioid receptor agonist-antagonist (activating к receptors, antagonizing some *μ* receptors) that can produce central analgesic and sedative effects (10, 11). It can be utilized for premedication, sedation, and postoperative pain management in pediatric patients. Furthermore, it also counteracts the adverse side effects of other opioids, such as itching or difficulty urinating (12). Nalbuphine exerts a weak inhibitory effect on respiration and can antagonize some μ receptors (13). We hypothesized that administering nalbuphine 5 min before the conclusion of the surgery could decrease the occurrence of EA, alleviate postoperative pain, and not cause any delay in extubation time or time to discharge from the PACU in pediatric patients. In order to achieve this objective, we conducted a prospective, randomized, and double-blind study. The purpose of the study was to examine if nalbuphine might effectively reduce EA in children who underwent tonsillectomy and adenoidectomy under sevoflurane general anesthesia.

## 2 Materials and methods

### 2.1 Study subjects

The study adhered to the guidelines established by the Helsinki Declaration and received approval from the Ethics Committee of Shanghai Children's Hospital (Ethics Approval No. 2023R029-E02). The parents of all the enrolled children signed an informed consent form. The study was registered with a clinical trial registration number ChiCTR2300070046 (registration date, March 31, 2023). The children who underwent adenoidectomy and tonsillectomy at Shanghai Children's Hospital from April 2023 to August 2023 were selected regardless of gender based on the following criteria: American Society of Anesthesiologists (ASA) physical status I or II, age 4–9 years, height 100–160 cm and weight 10–50 kg. The following patients were excluded from the study: those with hypertension, severe respiratory obstruction, liver and kidney dysfunction, neurological disorders, and asthma.

### 2.2 Randomization

The randomization process was conducted by unsealing an envelope with a unique serial number. The allocation sequence was constructed using a random permuted block randomization method. The research drugs were prepared by a nurse unaware of the investigation's details. All individuals involved, including investigators, anesthesiologists, surgeons, parents, and children, were unaware of the group they were assigned to.

### 2.3 Anesthesia methods

All children were fasted for 8 h and were not given any preoperative medications. Upon entering the operating room, anesthesia monitors were used to monitor vital signs, including electrocardiogram (ECG), blood pressure (BP), percutaneous pulse oxygen saturation (SpO_2_), and heart rate (HR). After an intravenous injection of 0.01 mg/kg atropine, general anesthesia was induced with 0.3 μg/kg sufentanil, 3 mg/kg propofol, and 0.1 mg/kg cisatracurium. Endotracheal intubation was executed using a video laryngoscope and mechanical ventilation with pressure control mode. The respiratory parameters were set as target tidal volume, 8–10 ml/kg; respiratory rate, 14–18 breaths/min; oxygen concentration, 0.6; oxygen flow rate, 2 L/min; positive end-expiratory pressure, 0. The end-tidal CO_2_ was maintained between 35 and 45 mmHg by adjusting the tidal volume. We used 2%–4% sevoflurane inhalation (beginning with 4% sevoflurane for 10 min, then 2%–3%) to maintain systolic blood pressure (SBP) within a range of 20% above or below the baseline value, and the BIS values were maintained between 40 and 60. If the decrease in the SBP exceeded 20% of the baseline value, 0.2 mg/kg ephedrine was intravenously injected. If HR < 60/min, 0.01 mg/kg of atropine was intravenously injected. Sevoflurane was discontinued 5 min before the conclusion of the procedure. At that time, the nalbuphine group received an intravenous injection of 0.2 mg/kg nalbuphine. On the other hand, the control group received an equivalent amount of normal saline by injection. Following the procedure, a dosage of 0.75 mg/kg ondansetron was given to avoid any occurrence of postoperative nausea and vomiting. The same surgeons performed the surgical procedures. The tracheal catheter was removed when the patients resumed spontaneous breathing with a tidal volume > 6 ml/kg and SpO_2_ > 94%. Patients were transferred from the operating room to the PACU.

### 2.4 Measurement

The extubating time (from the end of the surgery up until the removal of the tracheal catheter) and duration of PACU [from the end of the surgery to the Aldrete score (14) ≥9] were recorded. The SBP, diastolic blood pressure (DBP), and HR were monitored at 5-min intervals. The Face Legs Activity Cry and Consolability (FLACC) scales (15) and sedation scores were determined within 12 h after surgery at 1-h interval.

All drug-related postoperative complications, including EA, delayed awakening, nausea and vomiting, pruritus, and respiratory depression, were recorded. In the case of EA, the patient was intravenously injected with 1–2 mg/kg propofol. Tachycardia was defined as HR > 20% of the baseline value. Delayed awakening was defined as the patient's inability to open their eyes within 30 min after completion of the surgery. Respiratory depression was defined as SpO_2_ below 94% during nasal catheter oxygen inhalation of 3 L/min. The sedation level was assessed using the Ramsay sedation score (RSS) (16) (1) patient anxious, agitated, or restless; (2) patient cooperative, oriented, alert, and tranquil; (3) patient responds to commands; (4) asleep, but with rapid response to a light glabellar tap or loud auditory stimulus; (5) sleeping, with sluggish response to a light glabellar tap or loud auditory stimulus; (6), asleep, with no response. EA was defined as an RSS score of 1.

### 2.5 Statistical analysis

Statistical analysis was conducted using SPSS 20. The quantitative variables with normal distribution were expressed as mean ± standard deviation (SD) and were analyzed by *t*-test or analysis of variance (ANOVA). The Wilcoxon Mann–Whitney test examined the quantitative variables with a non-normal distribution. Categorical variables were analyzed using either the chi-square test or Fisher's exact test, and the results were presented in quantity or percentage. The primary outcome of this study was the incidence of EA. Secondary outcomes were pain scores, extubation time, and duration of PACU. A pilot study with 10 patients in each group showed that the incidence of EA was 30% and 10%, respectively. The incidence of EA was reduced by 66.7% in the nalbuphine group. A sample size of 60 patients was calculated using Power Analysis & Sample Size software 2020 (NCSS, LLC, Kaysville, UT, USA) to achieve an *α* of 0.05 and a power of 0.8. The sample size was increased to 65 to account for withdrawals from the study during the trial. Thus, a total of 130 patients were required. A value of *P *< 0.05 indicated statistical significance.

## 3 Results

### 3.1 Data of children

**Figure 1:**
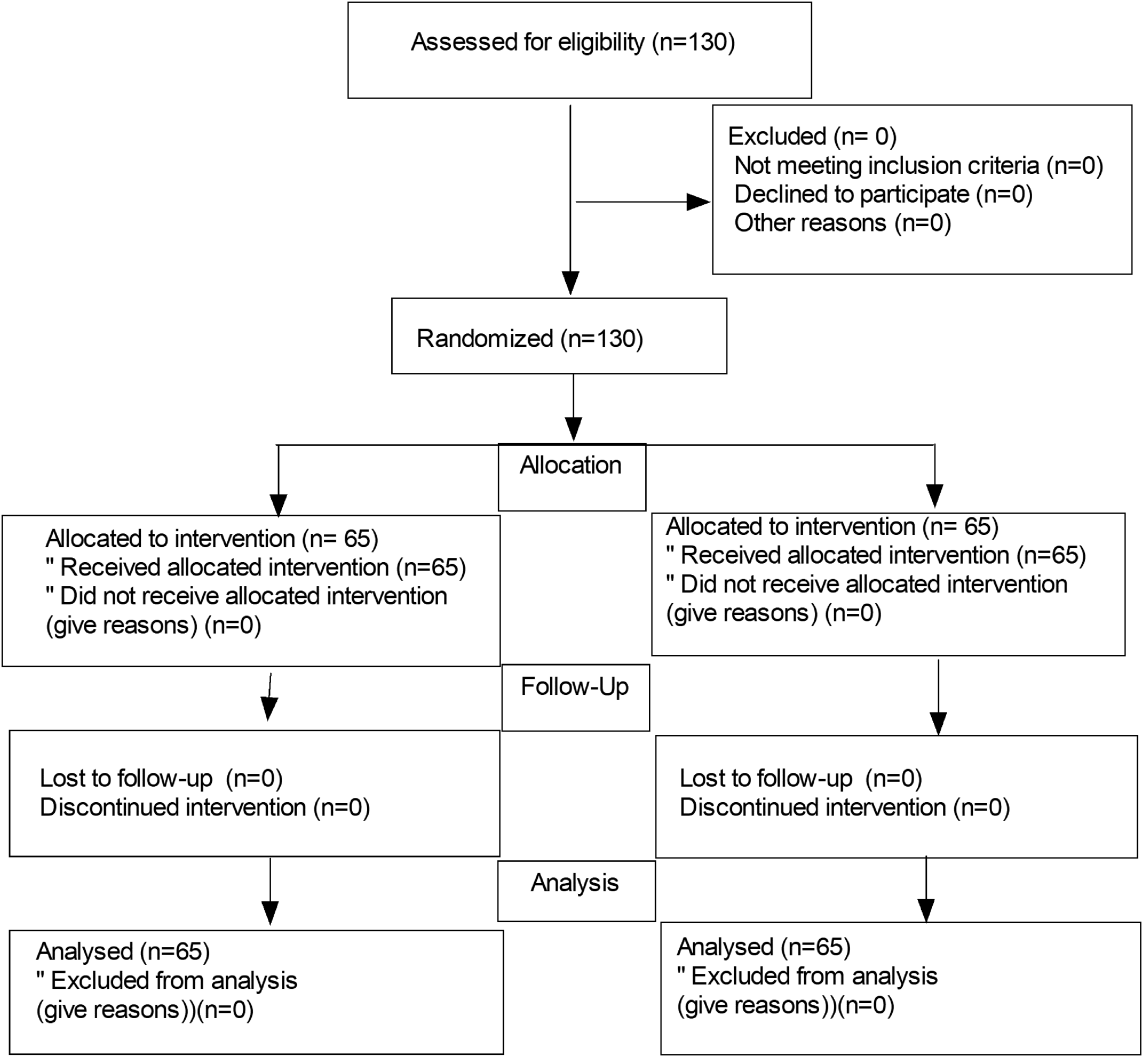
Flow diagram of the study.

One hundred and thirty pediatric patients were recruited, and then 130 patients were randomly divided into a nalbuphine group and a control group (*n* = 65 each). The flow diagram of the study is shown in Figure 1. There were no significant differences in age, weight, gender, duration of surgery, extubation time, time to eye-opening, and duration of PACU between the two groups (Table 1).

**Table 1:** Characteristics of the pediatric patients in both groups (*n *= 65).

Index	Nalbuphine group	Control group	*P*-Value
Age (years)	5.3 ± 1.3	5.5 ± 1.3	0.550
weight (kg)	20.6 ± 4.7	21.4 ± 4.5	0.321
Gender	36/29	31/34	0.380
Duration of operation (min)	19.9 ± 6.0	18.4 ± 4.3	0.109
Duration of anesthesia (min)	31.1 ± 6.6	30.7 ± 4.4	0.474
Extubating time (min)	12.9 ± 2.3	12.5 ± 3.3	0.337
Time to eye opening	24.0 ± 4.4	23.0 ± 5.6	0.269
Duration of PACU (min)	30.0 ± 5.1	29.6 ± 5.8	0.677

Date are expressed as mean ± SD or numbers. PACU, post-anesthesia care unit.

### 3.2 Hemodynamics

**Figure 2:**
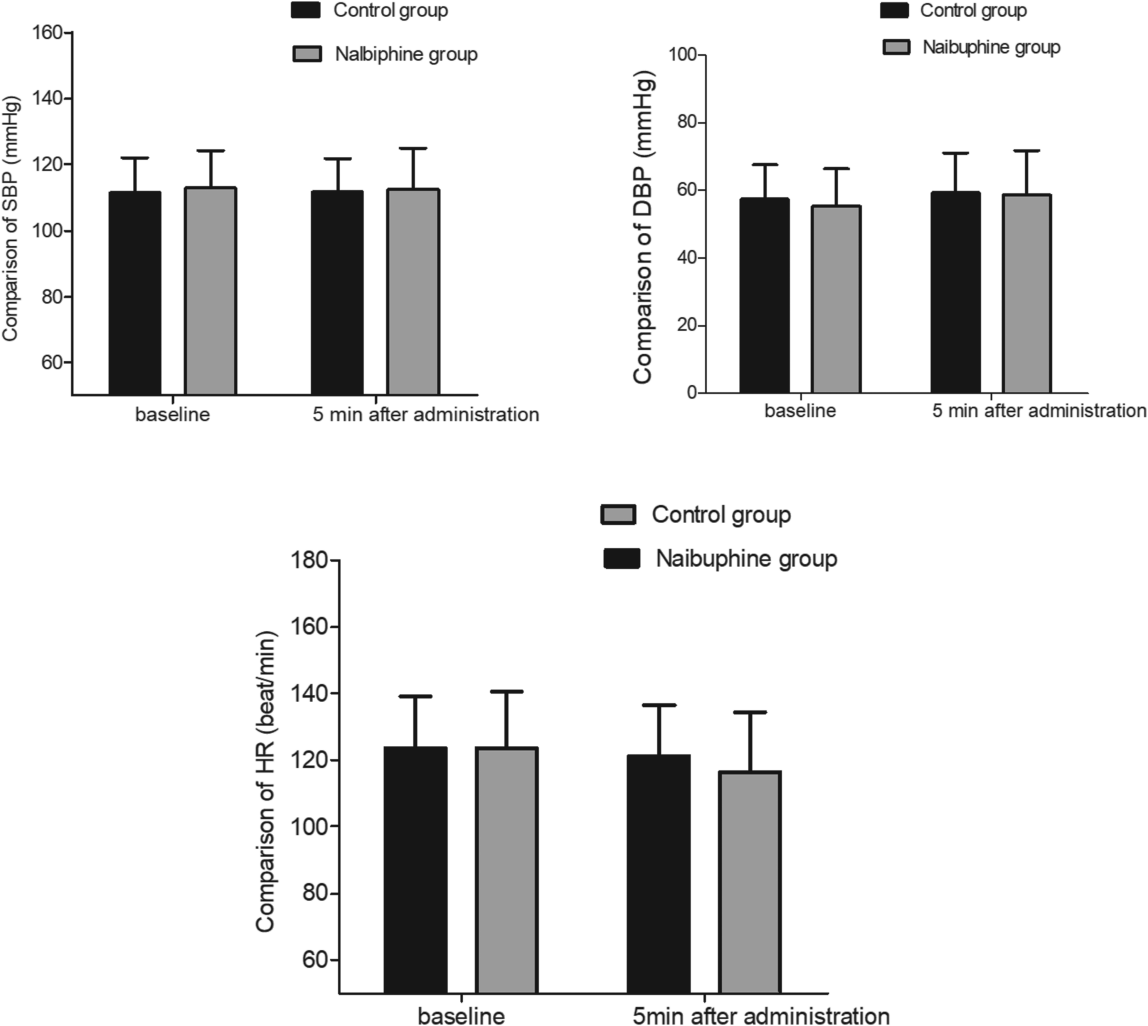
Comparison of hemodynamics between the two groups. There were no significant differences in hemodynamics between the two groups. SBP: systolic blood pressure, DBP: diastolic blood pressure, HR: heart rate.

The SBP, DBP, and HR after nalbuphine administration were not decreased obviously in the nalbuphine group (*P *> 0.05). No significant differences were observed in SBP, DBP, and HR values between the two groups 5 min after nalbuphine administration (*P *> 0.05, Figure 2).

### 3.3 Analgesic effect

**Figure 3:**
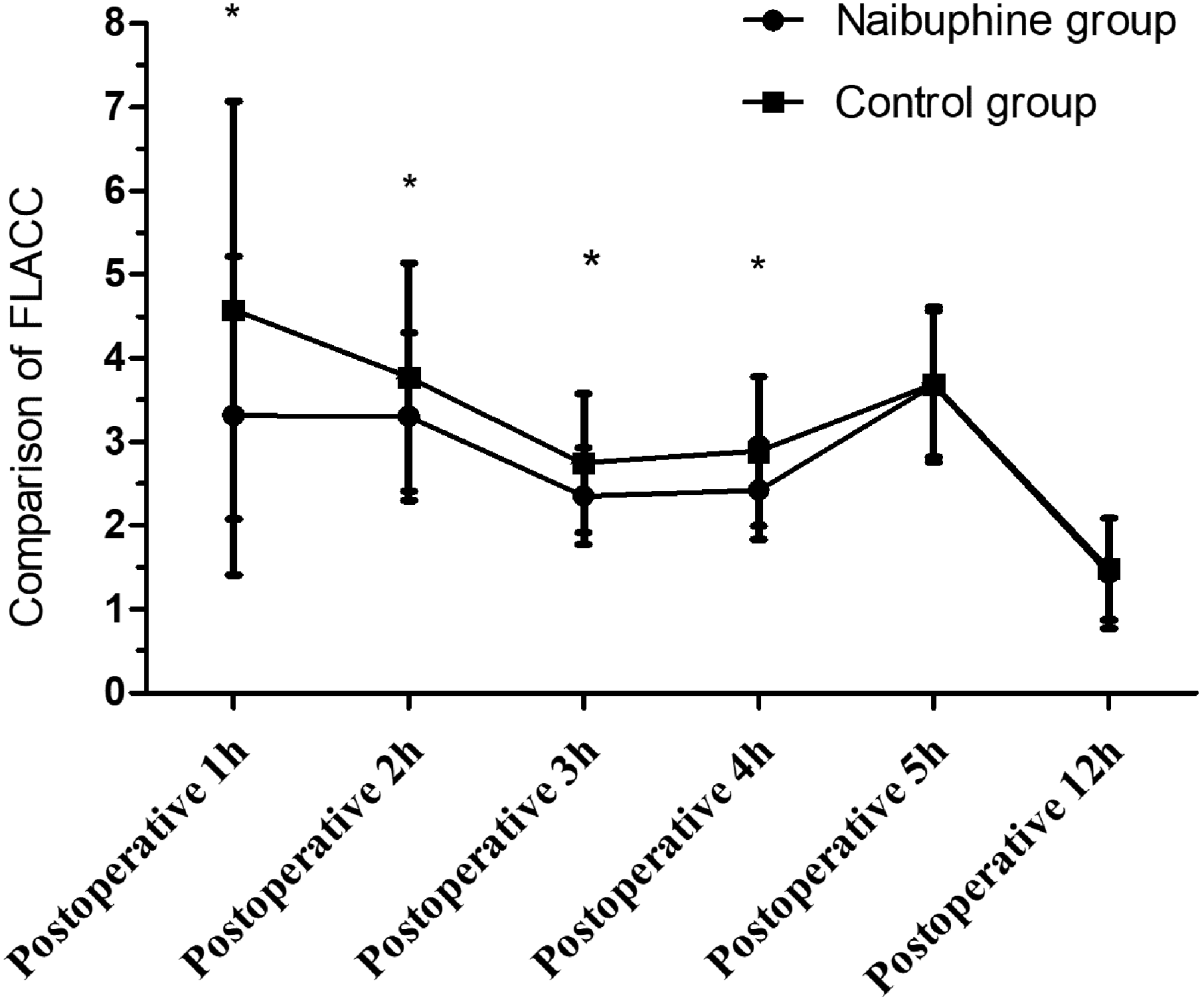
Comparison of FLACC scores between the two groups. FLACC scores within the first postoperative 4 h were lower in the nalbuphine group than in the control group. While FLACC scores during the 5–12 h postoperatively were similar between the two groups. *P < 0.05 compared to the control group using the Wilcoxon Mann–Whitney test. FLAAC: the Face Legs Activity Cry and Consolability scale.

During the first 4 h following surgery, the FLACC scores in the nalbuphine group were lower than those in the control group (*P *< 0.05). However, at postoperative hours 5 and twelve, the FLACC scores for the two groups were comparable (*P *> 0.05, Figure 3).

### 3.4 Adverse reactions

The incidence of EA was drastically decreased in the nalbuphine group compared with that in the control group **(**20% vs. 46.2%, *P *= 0.002**)**. However, the incidence of excessive sedation, delayed awakening, respiratory depression, pruritus, nausea, and vomiting was similar between the two groups (*P *> 0.05, Table 2).

**Table 2:** Complications in the two groups (*n *= 65).

Index	Nalbuphine group	Control group	*P*-Value
Emergence agitation	13	30	0.002*
Tachycardia	3	2	0.999
Respiratory depression	0	0	0.999
Nausea/vomiting	2	3	0.999
Excessive sedation	0	0	0.999
Delayed awakening	0	0	0.999
Pruritus	1	1	0.999

Date are expressed as numbers.

*P < 0.05.

## 4 Discussion

This study showed that preventive intravenous nalbuphine injection could lower the incidence of EA in young patients having ENT surgery without significantly extending the time spent in the PACU or during extubation.

EA is an essential issue in pediatric patients receiving general anesthesia. The origin of EA in children remains unclear. There are many reasons underlying the postoperative agitation observed following general anesthesia. Postoperative pain, inhalation of anesthetics, rapid recovery, surgical type (e.g., ENT surgery), hypoxia, and airway obstruction are all associated with the occurrence of postoperative agitation. Postoperative pain and discomfort are the leading causes of postoperative agitation in children (17, 18). Nalbuphine is an analgesic that produces analgesic effects by binding K and μ opioid receptors (11). Prophylactic 0.2 mg/kg nalbuphine treatment in this trial successfully decreased postoperative pain, which in turn reduced the incidence of EA. Furthermore, the 0.2 mg/kg nalbuphine injection did not prolong the extubation period, most likely because of its insignificant impact on the μ receptor-induced respiratory depression. We also found that nalbuphine did not cause an extension of the time to eye-opening and duration of PACU when administered 5 min before the end of surgery. This was due to the weak sedative effect of nalbuphine. He et al. (19) found that administration of 0.1 mg/kg nalbuphine effectively reduced the incidence of postoperative agitation without increasing recovery time, which was in line with the results of our study. In the present study, the extubating time was not prolonged as previously reported, possibly due to the weak respiratory depression produced by nalbuphine. The dose of nalbuphine (0.2 mg/kg) in our study was twice the dose of nalbuphine (0.1 mg/kg) in the previous research, but the results were similar between the two studies. The results of this study demonstrated that increasing nalbuphine dosage did not deteriorate the symptoms of respiratory depression. Nalbuphine induced a maximum level of sedation and respiratory depression (20).

In the present study, the FLACC scores in the nalbuphine group were lower than those in the control group within the first 4 h postoperatively. However, no statistically significant differences between the two groups were observed within 5–12 h postoperatively. It showed that nalbuphine at 0.2 mg/kg could provide adequate analgesia, lasting for 4 h. Besides, this study indicated that early pain would increase EA incidence. Liaqat et al. (21) found that the mean time for the requirement of rescue analgesics was 6.5 h in children who underwent inguinal herniotomy when given a single dose of nalbuphine (0.2 mg/kg) immediately after surgery. However, the duration of analgesia with nalbuphine was shorter in our study, possibly related to the type of surgery.

Nalbuphine at a dose of 0.2 mg/kg reduced the incidence of EA. By activating the к receptor, nalbuphine, an opioid receptor agonist-antagonist, promotes fatigue and analgesia. Nalbuphine's analgesic and sedative properties may help lower the EA incidence. On the other hand, neither group experienced significantly higher rates of respiratory depression, excessive sedation, delayed awakening, nausea, or vomiting. Nalbuphine failed to increase the incidence of postoperative complications, which is consistent with previous findings (19). Ali et al. (22) found that dexmedetomidine 0.3 μg/kg or propofol 1 mg/kg could reduce the incidence of EA compared with normal saline when administered 5 min before the end of surgery in children undergoing adenotonsillectomy under sevoflurane anesthesia, and dexmedetomidine 0.3 μg/kg was more effective than propofol 1 mg/kg in decreasing the incidence of EA. Their results were similar to ours.

The SBP, DBP, and HR showed negligible variations after nalbuphine administration in the nalbuphine group. As the incidence of nalbuphine-related adverse reactions is low, intravenous injection of nalbuphine may serve as an effective strategy to prevent EA.

## 5 Limitations

This study has some limitations. Firstly, the FLACC scales may not effectively assess the level of pain in young children who are unable to evaluate their suffering properly. Furthermore, the sample size is limited. Finally, the outcomes are influenced by the psychological and behavioral traits of the children. The age, sex, and pre-existing anxiety of the children affect the occurrence of EA.

## 6 Conclusions

In conclusion, the results of this study revealed that the prophylactic nalbuphine reduced the incidence of EA in pediatric patients receiving ENT surgery without increasing the extubating time and PACU duration.

## Data Availability

The raw data supporting the conclusions of this article will be made available by the authors, without undue reservation.

## References

[1] Goa KL Noble S Spencer CM. Sevoflurane in paediatric anaesthesia: a review. Paediatr Drugs. (1999) 1:127–53. 10.2165/00128072-199901020-00005 10937447

[2] Veyckemans F. Excitation and delirium during sevoflurane anesthesia in pediatric patients. Minerva Anestesiol. (2002) 68:402–5. 12029254

[3] Mason KP. Pediatric emergence delirium: a comprehensive review and interpretation of the literature. Br J Anaesth. (2017) 118:335–43. 10.1093/bja/aew477 28203739

[4] Kawai M Kurata S Sanuki T Mishima G Kiriishi K Watanabe T The effect of midazolam administration for the prevention of emergence agitation in pediatric patients with extreme fear and non-cooperation undergoing dental treatment under sevoflurane anesthesia, a double-blind, randomized study. Drug Des Devel Ther. (2019) 13:1729–37. 10.2147/DDDT.S198123 31190751 PMC6529617

[5] van Hoff SL O'Neill ES Cohen LC Collins BA. Does a prophylactic dose of propofol reduce emergence agitation in children receiving anesthesia? A systematic review and meta-analysis. Paediatr Anaesth. (2015) 25:668–76. 10.1111/pan.12669 25917689

[6] Ramlan AAW Pardede DKB Marsaban AHMS Hidayat J Peddyandhari FS. Efficacy of 0.5 mg/kg of propofol at the end of anesthesia to reduce the incidence of emergence agitation in children undergoing general anesthesia with sevoflurane. J Anaesthesiol Clin Pharmacol. (2020) 36:177–81. 10.4103/joacp.JOACP_257_19 33013031 PMC7480301

[7] Kim MS Moon BE Kim H Lee JR. Comparison of propofol and fentanyl administered at the end of anaesthesia for prevention of emergence agitation after sevoflurane anaesthesia in children. Br J Anaesth. (2013) 110:274–80. 10.1093/bja/aes382 23103775

[8] Kanaya A. Emergence agitation in children: risk factors, prevention, and treatment. J Anesth. (2016) 30:261–7. 10.1007/s00540-015-2098-5 26601849

[9] Li Q Fan J Zhang W. Low-dose esketamine for the prevention of emergency agitation in children after tonsillectomy: a randomized controlled study. Front Pharmacol. (2022) 13:991581. 10.3389/fphar.2022.991581 36605396 PMC9807658

[10] Jannuzzi RG. Nalbuphine for treatment of opioid-induced pruritus: a systematic review of literature. Clin J Pain. (2016) 32:87–93. 10.1097/AJP.0000000000000211 26650717

[11] Inan S Torres-Huerta A Jensen LE Dun NJ Cowan A. Nalbuphine, a kappa opioid receptor agonist and mu opioid receptor antagonist attenuates pruritus, decreases IL-31, and increases IL-10 in mice with contact dermatitis. Eur J Pharmacol. (2019) 864:172702. 10.1016/j.ejphar.2019.172702 31568781 PMC6913640

[12] Kubica-Cielińska A Zielińska M. The use of nalbuphine in paediatric anaesthesia. Anaesthesiol Intensive Ther. (2015) 47(3):252–6. 10.5603/AIT.2015.0036 26165241

[13] Liu YY Hsiao HT Wang JC Liu YC Wu SN. Effectiveness of nalbuphine, a *κ*-opioid receptor agonist and μ-opioid receptor antagonist, in the inhibition of I_Na_, I_K(M)_, and I_K(erg)_ unlinked to interaction with opioid receptors. Drug Dev Res. (2019) 80:846–56. 10.1002/ddr.21568 31301190

[14] Zhu W Sun J He J Zhang W Shi M. A randomized controlled study of caudal dexmedetomidine for the prevention of postoperative agitation in children undergoing urethroplasty. Front Pediatr. (2021) 29:658047. 10.3389/fped.2021.658047 PMC8513864 34660472

[15] Peng T Qu S Du Z Chen Z Xiao T Chen R. A systematic review of the measurement properties of face, legs, activity, cry and consolability scale for pediatric pain assessment. J Pain Res. (2023) 16:1185–96. 10.2147/JPR.S397064 37064956 PMC10094406

[16] Zhang W Li C. EC50 of epidural ropivacaine combined with dexmedetomidine for labor analgesia. Clin J Pain. (2018) 34:950–3. 10.1097/AJP.0000000000000613 29595529

[17] Zhong HY Deng XB Wang Z. Effects of fascia iliaca compartment block combined with general laryngeal mask airway anesthesia in children undergoing femoral fracture surgery: a randomized trial. J Pain Res. (2018) 11:2821–726. 10.2147/JPR.S177122 30519084 PMC6235337

[18] Ali I Alahdal M Xia H Moughrabi ASE Shiqian H Yao S. Ketofol performance to reduce postoperative emergence agitation in children undergoing adenotonsillectomy. Libyan J Med. (2020) 15:1688450. 10.1080/19932820.2019.1688450 31771436 PMC6882471

[19] He J Zhang L Tao T Wen X Chen D Zheng X Nalbuphine reduces the incidence of emergence agitation in children undergoing adenotonsillectomy: a prospective, randomized, double-blind, multicenter study. J Clin Anesth. (2023) 85:111044. 10.1016/j.jclinane.2022.111044 36566649

[20] Romagnoli A Keats AS. Ceiling effect for respiratory depression by nalbuphine. Clin Pharmacol Ther. (1980) 27:478–85. 10.1038/clpt.1980.67 7357806

[21] Liaqat N Dar SH. Comparison of single -dose nalbuphine versus tramadol for postoperative pain management in children: a randomized, controlled trial. Korean J Anesthesiol. (2017) 70:184–7. 10.4097/kjae.2017.70.2.184 28367289 PMC5370303

[22] Ali MA Abdellatif AA. Prevention of sevoflurane related emergence agitation in children undergoing adenotonsillectomy: a comparison of dexmedetomidine and propofol. Saudi J Anaesth. (2013) 7(3):296–300. 10.4103/1658-354X.115363 24015133 PMC3757803

